# Revisiting the halogen bonding between phosphodiesterase type 5 and its inhibitors

**DOI:** 10.1007/s00894-018-3897-z

**Published:** 2019-01-07

**Authors:** Wiktoria Jedwabny, Edyta Dyguda-Kazimierowicz

**Affiliations:** Department of Chemistry, Wrocław University of Science and Technology, Wrocław, Poland

**Keywords:** Inhibitors, Interaction energy, PDE5, Scoring

## Abstract

Halogenated ligands are nowadays commonly designed in order to increase their potency against protein targets. Although novel computational methods of evaluating the affinity of such halogenated inhibitors have emerged, they still lack the sufficient accuracy, which is especially noticeable in the case of empirical scoring functions, being the method of choice in the drug design process. Here, we evaluated a series of halogenated inhibitors of phosphodiesterase type 5 with ab initio methods, revealing the physical nature of ligand binding and determining the components of interaction energy that are essential for proper inhibitor ranking. In particular, a nonempirical scoring model combining long-range contributions to the interaction energy provided a significant correlation with experimental binding potency, outperforming a number of commonly used empirical scoring functions. Considering the low computational cost associated with remarkable predictive abilities of the aforementioned model, it could be used for rapid assessment of the ligand affinity in the process of rational design of novel halogenated compounds.

**Electronic supplementary material:**

The online version of this article (10.1007/s00894-018-3897-z) contains supplementary material, which is available to authorized users.

## Introduction

It has been recognized that halogen compounds (iodine, bromine, chlorine or even, to some extent, fluorine) interact with atoms possessing a lone electron pair (Lewis base, e.g., O, N, S atoms or *π*-electron systems) through so called halogen bonding, where the halogen atom acts as an acceptor (Lewis acid) [1–3]. Halogen bonding is related to the anisotropy of the electron density and the emergence of increased electrostatic potential, i.e., *σ*-holes [4, 5]. This electrostatically driven, directional, intermolecular interaction [6] has been successfully exploited in the drug development process [7–10], and is often found in biomolecular complexes [2, 11, 12]. Since commonly used empirical or knowledge-based docking and scoring approaches do not always properly account for the anisotropy of electron charge distribution of halogen residues, the quantum mechanical (QM) methods are usually required, when it comes to the modeling of halogen bonded systems [12–15]. Importantly, scoring of ligands bearing halogen atoms with empirical scoring functions is often not reliable [14, 16], although a progress in this field has been achieved, and novel empirical scoring functions dedicated to halogenated compounds have appeared [17–19].

As the QM-based methods are too computationally demanding to be applied in the drug design process [3, 17], reliable and fast scoring approaches are needed. Our non-empirical $E_{EL,MTP}^{(10)}+E_{Das}$ function [20], already validated with various protein-ligand complexes [21–23], might appear useful. Taking advantage of the long-range interaction energy terms including electrostatic atomic multipole expansion ($E_{EL,MTP}^{(10)}$) and approximate dispersion function (*E*_*Das*_), it constitutes a low cost approach that can be used in various biomolecular systems. The damped dispersion expression, *E*_*Das*_, has already been proven to successfully describe non-covalent interactions with atom-atom potentials fitted to reproduce the results of high-level quantum chemical calculations [24, 25]. Importantly, *E*_*Das*_ function appears to be an alternative to the commonly applied DFT-based damped dispersion corrections that might not be able to recover dispersion energies properly due to recently demonstrated unphysical damping occurring at small intermonomer distances [26]. However, available *E*_*Das*_ parameters have not covered halogen atoms [24, 25], restricting application of $E_{EL,MTP}^{(10)}+E_{Das}$ function to nonhalogenated compounds.

Novel *E*_*Das*_ parameters derived recently^1^ have enabled application of the $E_{EL,MTP}^{(10)}+E_{Das}$ model to scoring of halogen-substituted ligands. Preliminary tests of $E_{EL,MTP}^{(10)}+E_{Das}$ function augmented by halogen parameters demonstrated its favourable performance for the series of phosphodiesterase type 5 (PDE5) inhibitors. PDE5 enzyme catalyzes hydrolysis of cyclic guanosine monophosphate (cGMP), an intracellular second messenger molecule involved in multiple signaling pathways. Decreased degradation of cGMP due to PDE5 inhibition enhances its effects, leading to, e.g., relaxation of vascular smooth muscle tissue. PDE5 inhibitors (PDE5Is) are used in the treatment of pulmonary hypertension or erectile dysfunction (ED) [27]. ED therapy with PDE5 inhibitors, e.g., sildenafil (Viagra, Pfizer Inc., first generation PDE5Is) or avanafil (Stendra, Metuchen Pharmaceuticals, LLC., second generation PDE5Is) is preferred as the first-line medication [28]. Moreover, the number of various PDE5Is in dietary supplements has increased from 1 in 2003 to 69 in 2016 [28]. Recently, PDE5Is have been proposed as medicines for heart failure [29]. PDE5 has also been linked to tumor development regulation [30]. Due to clinical potential of PDE5 inhibition, development of higher affinity PDE5Is is of special interest. Understanding of the molecular basis of PDE5Is binding could significantly aid the rational design of novel inhibitors.

$E_{EL,MTP}^{(10)}+E_{Das}$ function complemented by novel halogen *E*_*Das*_ parameters has been validated using the set of PDE5 inhibitors, that involved monocyclic pyrimidinones with halogen bonding introduced to strengthen the binding affinity [31]. Here, we report nonempirical investigation of these compounds, providing detailed analysis of the physical basis of their interactions with PDE5. Furthermore, our $E_{EL,MTP}^{(10)}+E_{Das}$ approach is compared with a number of empirical scoring functions, including those recommended for halogen-bearing ligands (e.g., XBSF implemented in AutoDock VinaXB program [14]). Thermodynamical basis of binding of PDE5 inhibitors studied here have been characterized experimentally by Ren et al. [32]. The experimental results discussed therein were accompanied by theoretical analysis covering only a limited representation of the binding site, leaving a room for further and more complete computational examination, which we have therefore exploited. In particular, calculations for only 3 out of 5 compounds were performed by Ren et al. [32], whereas herein we report computational results for all inhibitors.

Our recent findings [23, 33] suggest that interaction energy calculations should be accompanied by assessment of the solvation effects, since the considerable differences in the solvation free energy might affect the inhibitory activity ranking produced by herein applied theoretical models. As the latter account only for the enthalpic contribution to the binding free energy, their applicability requires consistency of free energy of solvation (Δ*G*_*solv*_) among the analyzed set of inhibitors. It could be especially important in the case of halogen-bearing ligands [34]. Accordingly, we complement our analysis of halogenated PDE5 inhibitors by the report on the related solvation effects.

## Methods

### Preparation of protein-inhibitor complexes

**Fig. 1:**
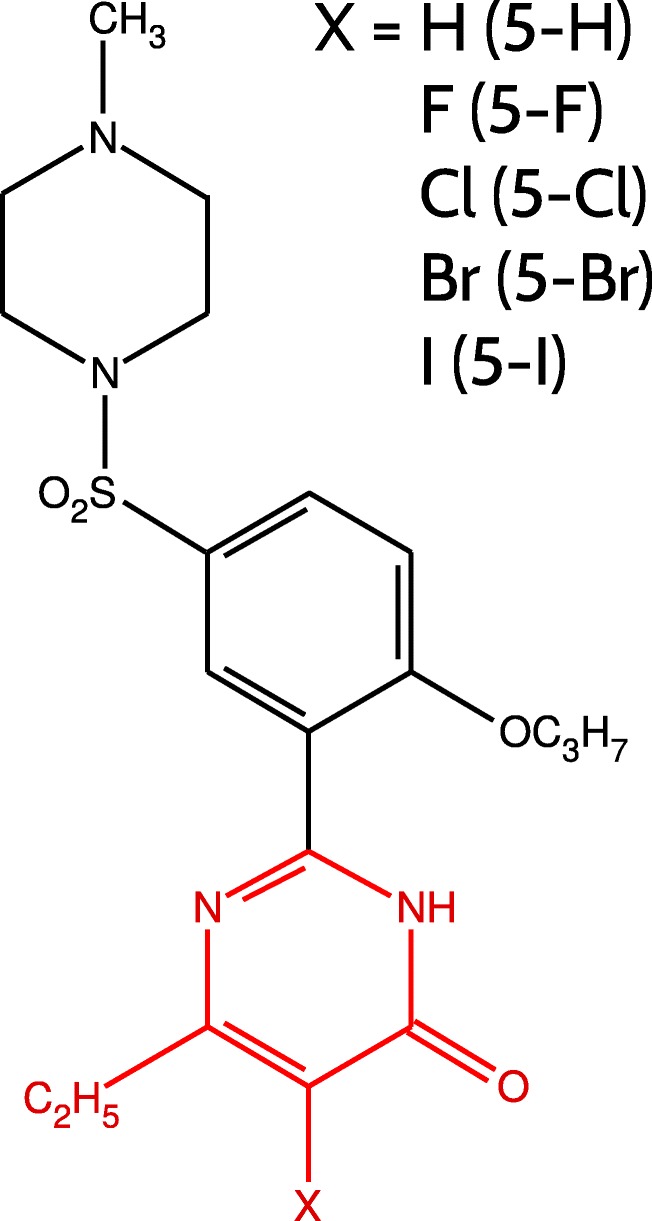
The structures of PDE5 inhibitors. Binding energy calculations were performed for the part of the structure marked in red

A set of 5 PDE5 inhibitors developed by Xu et al. [31] is examined with ab initio and empirical methods to analyze the effect of the halogen substitution on the binding affinity. Crystal structures of PDE5-inhibitor complexes are available via the following PDB codes: 4OEX, 4OEW [32], 3SIE, 3SHY, 3SHZ [31] (resolutions of 2.14, 2.44, 1.93, 2.65, and 2.45 Å, respectively). The inhibitor compounds share a common pyrimidinone scaffold. The only difference between these structures is the substitution at the 5-position of the pyrimidinone ring with a halogen or hydrogen atom (Fig. 1).

Optimal hydrogen bonding was determined with Propka [35–38], implemented in Maestro. Due to insufficient resolution of the most of the crystal structures (e.g., 3SHY complex with 2.65Å resolution), all protein-inhibitor structures were solvated with TIP3 water model [39] and optimized in Charmm program [40] (version c36b1). Both Charmm General Force Field v. 2b7 [41] and Charmm22 All-Hydrogen Force Field [42–44] parameter files were used. Missing parameters for inhibitor structures were generated with CGenFF interface at http://cgenff.paramchem.org [41, 45–47] (interface version 1.0.0). All amino acid residues further than 10 Å from each inhibitor were kept frozen throughout 1000 steps of steepest descent followed by conjugate gradient optimization until root mean square gradient of 0.01kcal ⋅mol^− 1^ ⋅ Å was reached.

**Fig. 2:**
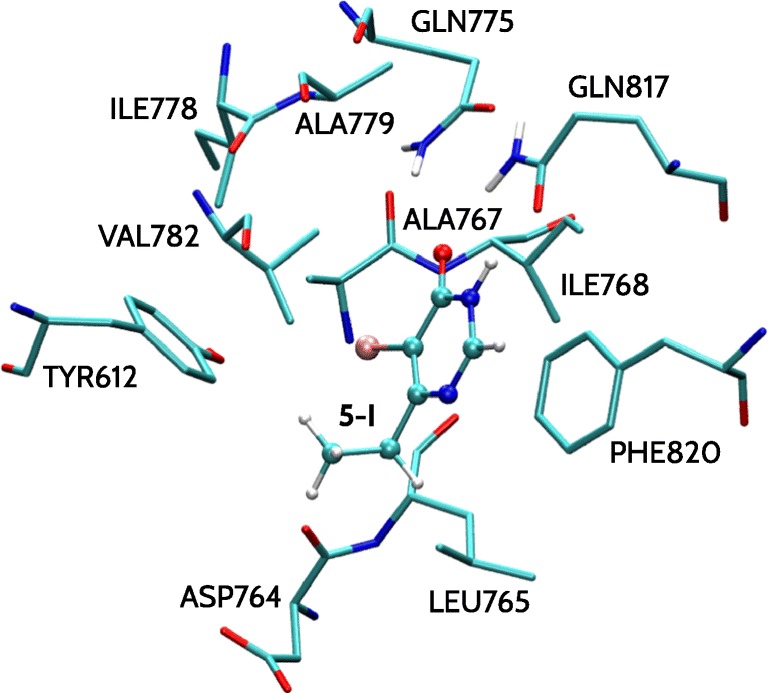
PDE5 binding site representation in complex with the model of **5-I** inhibitor

To investigate the influence of the substitution on the activity of the inhibitors, the inhibitors were truncated, as the common scaffold was positioned similarly in the case of all complexes. Therefore, only the pyrimidinone substituted at 5-position (marked in red in Fig. 1) was taken into account during the calculations. All amino acid residues within 5 Å of the substituent at pyrimidinone 5-position were selected to serve as PDE5 binding site model. The latter included 11 amino acid residues (Tyr612, Asp764, Leu765, Ala767, Ile768, Gln775, Ile778, Ala779, Val782, Gln817, Phe820; see Fig. 2). Dangling bonds resulting from truncation of the inhibitor structures and cutting the amino acid residues from the protein structure were filled with hydrogen atoms minimized in Schrödinger Maestro [48] program using Opls 2005 force field [49].

### Interaction energy calculations

Interaction energy between PDE5 binding site representation and each inhibitor was calculated within Hybrid Variation-Perturbation Theory (HVPT) [50, 51] decomposition scheme as the sum of binding energy values obtained for amino acid residue-inhibitor dimers. Each amino acid residue included in particular dimers was considered separately except for residues linked by a peptide bond, i.e., Asp764 and Leu765, Ala767 and Ile768, as well as Ile778 and Ala779 residues. Interaction energy calculations were carried out with GAMESS program [52] using polarized triple zeta valence basis set of Ahlrichs et al. (def2TZVP) [53, 54] and the corresponding effective core potential (ECP) on iodine atoms. Counterpoise correction was applied [55].

In HVPT, the Møller-Plesset second-order interaction energy (*E*_*MP*2_) is partitioned into the multipole electrostatic ($E_{EL,MTP}^{(10)}$), penetration ($E_{EL,PEN}^{(10)}$), exchange ($E_{EX}^{(10)}$), delocalization ($E_{DEL}^{(R0)}$) and correlation ($E_{CORR}^{(2)}$) terms:

$$ E_{MP2} = \underbrace{E_{EL,MTP}^{(10)}}_{R^{-n}} \!+ \underbrace{E_{EL,PEN}^{(10)} + E_{EX}^{(10)} + E_{DEL}^{(R0)}}_{exp(-\alpha R)} + \underbrace{E_{CORR}^{(2)}}_{R^{-n}}. $$

Interaction energy components introduced above can be grouped into long- and short-range energy terms that vary with the intermolecular distance *R* as *R*^−*n*^ or *exp*(−*aR*), respectively. The electrostatic multipole component of the binding energy, $E_{EL,MTP}^{(10)}$, was assessed with the Cumulative Atomic Multipole Moment (CAMM) expansion [56]. The latter is truncated at *R*^− 4^ term and computed using the correlated wave-function. The first-order electrostatic energy, $E_{EL}^{(10)}$, is equal to the sum of the penetration term, $E_{EL,PEN}^{(10)}$, and the $E_{EL,MTP}^{(10)}$ energy. Similarly, the first-order Heitler-London energy, *E*^(10)^, is obtained by adding the first-order electrostatic energy to the exchange component, $E_{EX}^{(10)}$. Subsequently, the higher order delocalization energy, $E_{DEL}^{(R0)}$, which comprises classical induction and charge transfer terms, is the difference between the counterpoise-corrected self-consistent field variational energy, *E*_*SCF*_, and the first-order Heitler-London energy, *E*^(10)^. Finally, the correlation term, $E_{CORR}^{(2)}$, accounting primarily for the dispersion, exchange-dispersion, and intramolecular correlation contributions, is obtained as the difference of the second-order Møller-Plesset interaction energy, *E*_*MP*2_, and converged SCF energy, *E*_*SCF*_. The $E_{EL,MTP}^{(10)}+E_{Das}$ energy values were calculated with a novel revision of the dispersion function, *E*_*Das*_.2

Empirical scoring was performed with several scoring functions. GoldScore, ChemScore, ChemPLP, and Astex Statistical Potential (ASP) functions, implemented in Gold 5.6.3 [57], were employed, with a spherical grid comprising amino acid residues within 10 Å radius from the point of origin, defined as the pyrimidinone 5-position. Further, ChemPLP and PLP scoring functions from Plants [58] docking program were used with a 10 Å radius sphere centered on the ligand. Since ChemPLP function is present in both Gold and Plants, it was assigned a subscript indicating its origin, namely ChemPLP_*G*_ (Gold) and ChemPLP_*P*_ (Plants). Despite sharing the same name, both functions are defined differently: ChemPLP_*G*_ combines ChemScore hydrogen bonding term with linear potentials for modeling the van der Waals and repulsive terms [59], while ChemPLP_*P*_ is based on the piecewise linear potential (PLP) scoring function [58]. Importantly, ChemPLP_*G*_ score is given in arbitrary units with higher score indicating more potent inhibitor, whereas ChemPLP_*P*_ yields the scoring results in kcal ⋅mol^− 1^ units (lower score indicates more potent inhibitor). GlideSP (standard precision) [60] and GlideXP (extra precision) [61] scoring functions from Schrödinger Glide program (revision 2018-1) [62] were also applied with a 10 Å grid centered on the ligand. XBSF scoring function, implemented in AutoDock VinaXB [14] and designed especially for halogenated ligands, was also employed for the comparison. Scoring was carried out with 10 Å cubic grid centered on the inhibitor.

In all docking programs full structures of protein-inhibitor complexes, optimized as described in *Preparation of protein-inhibitor complexes* section, were used for scoring of the existing poses of truncated inhibitor structures. In particular, no docking was performed, as the compounds re-docking would affect the results, precluding the comparison of the performance of both empirical and non-empirical scoring. __________ ^2^Unpublished results (2018)

### Assessment of solvation effects

The solvation free energy (Δ*G*_*solv*_) for each inhibitor was calculated in Gaussian09 with Polarizable Continuum Model (PCM) at the MP2/def2TZVP level of theory as single-point calculations performed for the inhibitor structures used throughout the computational protocol of interaction energy calculation. Integral equation formalism variant (IEFPCM) [63–65] was applied along with ExternalIteration [66, 67], DoVacuum, and SMD [68] options.

### Evaluation of the results

The performance of each scoring model was estimated with Pearson correlation coefficient (*R*) calculated with respect to the experimentally determined inhibitory activity reported by Ren et al. [32]. Dissociation constant *K*_*d*_ was chosen as a reference, since all thermodynamical data provided by Ren et al. [32] were obtained with the recombinant catalytic domain of human PDE5, whereas IC_50_ values referred to the full-length of rabbit PDE5. As a further performance measure, the statistical predictor *N*_*pred*_, representing the success rate of prediction of relative affinities, was applied. *N*_*pred*_ is calculated among all pairs of inhibitors as the percentage of concordant pairs with relative stability of the same sign as in the reference experimentally determined binding potency [69]. To enable comparison with the non-empirical interaction energy results, assigning lower binding energy values to more potent inhibitors, the scoring functions with higher score implying the greater binding potency were assigned the opposite values of the calculated correlation coefficient.

## Results and discussion

### Non-empirical models of PDE5 inhibitors binding

PDE5 binding site model considered herein includes 11 amino acid residues surrounding the inhibitor in the vicinity of 5 Å within the atom occupying pyrimidinone 5-position (Fig. 2). Asp764 is the only charged residue within this set. Except for Tyr612, Gln775, and Gln817, the remaining neutral residues are nonpolar. Total binding energies calculated by summing up the values characterizing individual amino acid residue-inhibitor dimers are given in Table 1. The corresponding *E*_*MP*2_ pairwise interaction energy values are provided in the Supplementary Material.

**Table 1:** Total PDE5-inhibitor interaction energy^*a*^ at the consecutive levels of theory

Inhibitor	$p\mathrm {K}_{\mathrm {d}}{~}^{b}$	${E}_{EL,MTP}^{(10)}$	${E}_{EL}^{(10)}$	*E* ^(10)^	*E*_*SCF*_	*E*_*MP*2_	${E}_{EL,MTP}^{(10)}+E_{Das}$
5-I	6.82	− 19.6	− 36.5	10.4	− 5.1	− 33.4	− 55.1
5-Br	6.38	− 19.7	− 36.7	11.9	− 3.2	− 30.1	− 52.9
5-Cl	6.12	− 17.0	− 30.2	4.5	− 7.7	− 30.1	− 46.5
5-H	5.84	− 19.4	− 35.3	5.6	− 7.9	− 28.7	− 47.6
5-F	5.76	− 18.1	− 32.4	5.9	− 6.8	− 26.7	− 45.7
R^*c*^		− 0.45	− 0.54	0.75	0.66	− 0.95	− 0.93
$N_{pred}{~}^{d}$		70.0	70.0	40.0	30.0	100.0	90.0

^a^ In units of kcal ⋅ mol^− 1^

^b^ K_*d*_ values are taken from Ref. [32]

^c^ Correlation coefficient between the energy obtained at a given level of theory and the experimental inhibitory activity

^d^ Percentage of successful predictions [%]

Negative values of total interaction energy calculated at the reference MP2 level of theory, *E*_*MP*2_, indicate the favorable interaction with PDE5 binding site. The prevalent contribution to binding energy at MP2 level of theory appears to be due to electrostatic interaction, $E_{EL}^{(10)}$ (Table 1). The positive values of the first-order Heitler-London energy, *E*^(10)^, imply the overestimated repulsion, as the short-range exchange contribution, $E_{EX}^{(10)}$, is particularly sensitive to any structural inaccuracies [70]. Accounting for the subsequent delocalization term, $E_{DEL}^{(R0)}$, restores the stabilizing nature of the resulting *E*_*SCF*_ interaction energy (Table 1), however, the proper ranking of inhibitors is established only after including the correlation contribution, $E_{CORR}^{(2)}$. Except for **5-Br** and **5-Cl** inhibitors, featuring the same value of *E*_*MP*2_ binding energy, the ranking of PDE5 inhibitors resulting from interaction energy calculated at MP2 level of theory (Table 1) is in agreement with experimental data [32].

To quantify the performance of particular interaction energy models, the relationship between the binding energy calculated at the subsequent levels of theory and the experimentally determined dissociation constants [32] was expressed by means of the Pearson correlation coefficient (*R*) and the success rate of prediction of relative inhibitory potency (*N*_*pred*_; see Table 1). The experimental binding affinities are reproduced only when dispersion is accounted for, i.e., at the *E*_*MP*2_ (*R* = − 0.95,*N*_*pred*_ = 100%) or $E_{EL,MTP}^{(10)}+E_{Das}$ (*R* = − 0.93,*N*_*pred*_ = 90%) levels of theory. In fact, the predictive ability of the $E_{EL,MTP}^{(10)}+E_{Das}$ model is essentially due to the dispersion contribution, *E*_*Das*_. As demonstrated by low values of *R* correlation coefficients, neither multipole electrostatic nor the first-order electrostatic interaction energy alone provide reliable predictions of the binding affinity in terms of the correlation with the experiment (*R* = − 0.45 and − 0.54 for $E_{EL,MTP}^{(10)}$ and $E_{EL}^{(10)}$, respectively; Table 1). *N*_*pred*_ values associated with these levels of theory (70% in both cases) are lower compared to the models accounting for the dispersion interactions, mostly due to the underestimation of the **5-Cl** inhibitor affinity.

Binding energies calculated at *E*^(10)^ level of theory anti-correlate with the experimental inhibitory activity, i.e., higher repulsion is associated with stronger inhibitors (Table 1). Due to exponential dependence of $E_{EX}^{(10)}$ contribution (accounted for at the *E*^(10)^ level of theory) on the interatomic distance, any minor structural deficiencies resulting from, e.g., insufficiently accurate starting geometries, significantly affect this particular interaction energy term [20, 70]. The extent to which $E_{EX}^{(10)}$ term is altered seems to be influenced by the overall binding strength, as the more potent ligands tend to experience higher repulsion originating from overestimated $E_{EX}^{(10)}$ contribution. In consequence, delocalization energy, $E_{DEL}^{(R0)}$, that contributes to the subsequent level of theory, namely *E*_*SCF*_, is not able to recover the proper inhibitory activity trend, as *E*_*SCF*_ remains anti-correlated with respect to the experimental ligand affinity (Table 1). Finally, the inverse ranking of PDE5 inhibitors is overcome with $E_{CORR}^{(2)}$ contribution and the resulting *E*_*MP*2_ energy provides a reasonable estimate of the inhibitory activity. The important role of the correlation term appears to be in agreement with the results of Riley et al. [71] demonstrating that halogen bonding involves the interplay between electrostatic and dispersion forces. As already emphasized, the performance of the approximate $E_{EL,MTP}^{(10)}+E_{Das}$ model, accounting for long-range interaction energy terms only, is comparable to the predictive capabilities of *E*_*MP*2_ binding energy. However, the computational cost of $E_{EL,MTP}^{(10)}+E_{Das}$ model is equivalent to force field calculations [20], as it scales with the square number of atoms, *O*(*A*^2^), while computational scaling of *E*_*MP*2_ energy is described by the fifth power of the number of atomic orbitals, *O*(*N*^5^).

The higher the atomic number of the substituting halogen (Cl, Br, I), the stronger the interaction with the protein, as described by nonempirical $E_{EL,MTP}^{(10)}+E_{Das}$ model. This is in agreement with other available studies of halogen series, where it has been shown that the strength of the halogen bond increases with the increasing polarizability of the halogen atoms [72]. In particular, the increased halogen atom polarizability has been related to the magnitude of *σ*-hole potential [73, 74]. At the MP2 level of theory, the interaction energy values of both **5-Cl** and **5-Br** inhibitors are roughly the same, probably due to the incorporation of the short-range contributions, especially $E_{EX}^{(10)}$. As a result, accounting for delocalization and correlation terms improves the overall performance of the *E*_*MP*2_ energy, but the binding affinities of the aforementioned compounds are still more similar than expected.

### Solvation effects

In our recent work on protein-protein interaction (PPI) inhibitors [23, 33], Δ*G*_*solv*_ has emerged as an important factor determining whether significant correlation between relative interaction energy values and experimental results could be obtained. Estimation of binding affinity with interaction energy-based models is justified only if the remaining contributions to binding free energy are essentially constant across the inhibitor series. Relatively high standard deviation (SD) values of solvation free energy characterizing a set of ligands indicate that no predictions could be made based on the interaction energy alone. Accordingly, any correlation with the experimental binding affinity other than coincidental should not be expected. To overcome this issue, the inhibitor set could be limited to compounds featuring similar Δ*G*_*solv*_ values.

As for the analyzed PDE5 inhibitors, the correlation between experimental affinity and *E*_*MP*2_ or $E_{EL,MTP}^{(10)}+E_{Das}$ binding energy already is significant. Nevertheless, it seemed important to verify solvation effects here as well, especially given the findings of Fanfrlik et al. [34], where binding energy of halogenated ligands yielded no correlation with experiment unless solvation energy was accounted for. The relatively low SD values of Δ*G*_*solv*_ calculated for PDE5 inhibitors with SMD model (Table 2) further support the predictive abilities of interaction energy-based models tested herein. The coincidence of low Δ*G*_*solv*_ standard deviation and the significant *R* correlation coefficient values for PDE5 inhibitors is in line with our previous analyses (see Refs. [23] and [33]), showing that in order to obtain correlation for the interaction energy, the associated solvent effects estimated with SMD model need to be relatively similar within the ligand set.

**Table 2:** Solvation free energy (Δ*G*_*solv*_) of PDE5 inhibitors along with the electrostatic (Δ*G*_*solv*,*el*_) and non-electrostatic (Δ*G*_*solv*,*non*−*el*_) contributions.^*a*^

Inhibitor	Δ*G*_*solv*_	Δ*G*_*solv*,*el*_	Δ*G*_*solv*,*non*−*el*_
5-I	− 8.6	− 11.7	3.2
5-Br	− 7.4	− 10.2	2.8
5-Cl	− 7.2	− 10.7	3.5
5-H	− 8.3	− 11.9	3.6
5-F	− 6.9	− 11.0	4.1
*S* *D* ^*b*^	0.73	0.72	0.46

^a^ In units of kcal ⋅mol^− 1^

^b^ Standard deviation calculated for the Δ*G*_*solv*_ energy values; in units of kcal ⋅mol^− 1^

As reported by Ren et al. [32], entropy contribution related to **5-F** inhibitor is substantially higher than for the remaining compounds (such observations are also described in the literature [75, 76]). In fact,the entropy term characterizing **5-F** compound exceeds the corresponding enthalpic term, while the latter constitutes the main binding free energy contribution for the remaining PDE5 inhibitors within this set [32]. Interestingly, this is not reflected in our solvation energy calculations, where **5-F** compound is associated with the lowest Δ*G*_*solv*_ value compared to the remaining inhibitors (see Table 2). Moreover, low SD values calculated for the analyzed PDE5Is indicate similar solvation effects across the series of ligands. Possibly, the methods for computing the solvation free energy of ligands are only approximate, yielding Δ*G*_*solv*_ results that suffer from significant errors [77–79], and, as a result, they do not represent the experimental results accurately enough.

### Insights into the nature of the PDE5Is’ binding

**Fig. 3:**
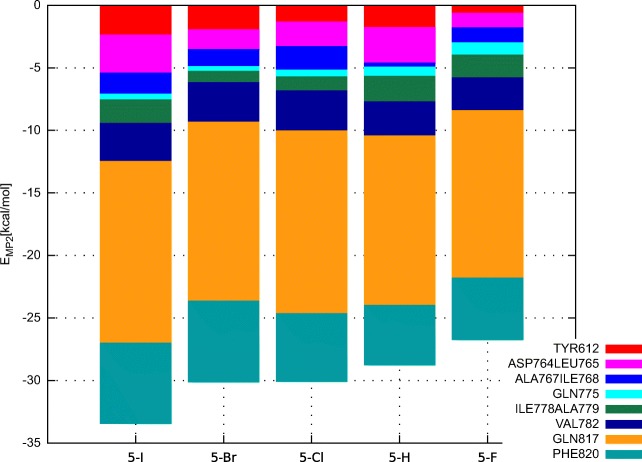
Contribution of individual amino acid residues or residue pairs to *E*_*MP*2_ interaction energy of PDE5 inhibitors

*E*_*MP*2_ binding energy values obtained for individual amino acid residues or residue pairs (Asp764-Leu765, Ala767-Ile768, and Ile778-Ala779) are shown in Fig. 3. The highest interaction energy values are due to Gln817 residue (− 13.4 to − 14.6kcal ⋅mol^− 1^, Table S1 in the Supplementary Material). Phe820 residue also contributes significantly to the overall binding energy (− 4.7 to − 6.4kcal ⋅mol^− 1^). Interestingly, the interaction with Tyr612 residue, that seems to be halogen bonded to the inhibitor molecules, is of moderate strength (− 0.7 to − 2.4kcal ⋅mol^− 1^). Apparently, interactions with the remaining closely positioned amino acid residues (Gln817, Phe820, Val782, Asp764, and Leu765; Fig. 3) contribute together to the overall binding strength. In this case, focusing on a particular interaction (i.e., halogen/hydrogen bonding with Tyr612), while neglecting the contributions arising from the other nearby residues during novel inhibitors design should be reconsidered. In particular, our results do not support the decreased binding strength in **5-I**-Tyr612 complex, accompanied by enhanced interaction with a buried water molecule, as observed by Ren et al. [32]. Further discussion on these differences is provided in the Supplementary Material.

As mentioned in the previous section, Ren et al. [32] reported substantial difference in the experimentally derived enthalpic/entropic contribution to the binding of **5-F** inhibitor, as compared to the remaining compounds. It was suggested that the low enthalpic term of **5-F** compound could be due to the electron withdrawal from the pyrimidinone ring upon the fluorine substitution, which probably results in reduced interactions with PDE5 amino acid residues, e.g., Phe820 or Val782 [32]. This was not confirmed by computational results reported herein (see Fig. 3 and Table S1 in the Supplementary Material), as binding of **5-F** due to Phe820 and Val782 residues is of a similar magnitude as in the case of **5-H** ligand.

Another possible reason pointed out by Ren et al. [32] for the decreased enthalpic contribution of **5-F** inhibitor involved weakening of the bond between Tyr612 and Gln817 residues and **5-F** compound. As shown in Fig. 3, **5-F**–Tyr612 interaction amounting to − 0.7kcal ⋅mol^− 1^ is indeed weaker than for the remaining compounds, featuring *E*_*MP*2_ interaction energy within the range of − 1.3 to − 2.4kcal ⋅mol^− 1^ (see Table S1 in the Supplementary Material). However, **5-F**–Gln817 *E*_*MP*2_ contribution is comparable to the corresponding value characterizing **5-H** compound (− 13.4 and − 13.5kcal ⋅mol^− 1^, respectively). Another weaker interaction of the **5-F** compound is the one with the Asp764-Leu765 residue pair (see Table S1 in Supplementary Material for details). It seems that the latter, together with the Tyr612 interaction, contribute to the somewhat smaller enthalpic term of **5-F** inhibitor. Weakening of the interaction upon fluorine substitution might be due to prevalent negative electrostatic potential characterizing fluorine atoms covalently bonded to aromatic systems [80]. Nevertheless, the difference in the enthalpic/entropic contribution of **5-F** compound is not as pronounced as one could expect given the experimental data.

### Empirical scoring

**Fig. 4:**
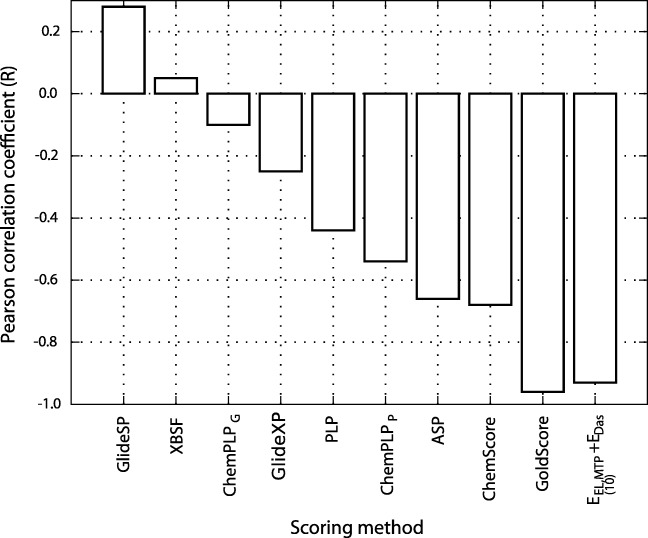
Pearson correlation coefficients obtained for the empirical scoring functions and nonempirical $E_{EL,MTP}^{(10)}+E_{Das}$ model

Scoring functions applied for the evaluation of PDE5Is included GoldScore, ChemPLP (ChemPLP_*G*_), ChemScore, and Astex Statistical Potential (ASP) from Gold 5.6.3, ChemPLP (ChemPLP_*P*_), and PLP scoring functions from Plants, XBSF from VinaXB, as well as GlideSP and GlideXP from Glide. Correlation coefficients associated with all these methods are compared in Fig. 4. The values of each empirical score obtained for PDE5 inhibitors, along with the corresponding correlation coefficients and *N*_*pred*_ values, are provided in Table S2 in the Supplementary Material.

Out of the nine scoring functions tested herein, only GoldScore function was capable of ranking the PDE5 inhibitors in sufficient agreement with the experimental inhibitory activity (*R* = − 0.96,*N*_*pred*_ = 90%, see Fig. 4). In most cases, the affinity of **5-Cl** and/or **5-F** inhibitor is severely overestimated (see Table S2 in Supplementary Material). This might indicate that the majority of the currently used scoring functions lack the ability to properly describe halogen interactions, and there still is a room for improvement. Overall, the performance of scoring functions appear to be different depending on the system studied [23] and there is no apparent choice of an appropriate scoring approach to be used with a certain protein-ligand system. However, our nonempirical $E_{EL,MTP}^{(10)}+E_{Das}$ model has been shown to possess the satisfactory predictive abilities for a variety of enzyme-inhibitor complexes [20, 21] including the PPI inhibitors [22, 23, 33]. Our current results seem to extend the range of application of $E_{EL,MTP}^{(10)}+E_{Das}$ model to halogen-bearing compounds.

## Summary

In this work, a congeneric series of halogenated PDE5 inhibitors, sharing a common pyrimidinone scaffold, was evaluated with the ab initio HVPT energy decomposition scheme, followed by the analysis with an approximate $E_{EL,MTP}^{(10)}+E_{Das}$ model, including only multipole electrostatic and dispersion interaction energy terms. The latter method of the assessment of the inhibitory activity was compared to a number of empirical scoring approaches.

The interaction energy calculated for such a model accurately reproduces the experimentally derived affinities, as the correlation coefficients with respect to experimental inhibitory activity are equal to − 0.95 and − 0.93 for the reference *E*_*MP*2_ and nonempirical $E_{EL,MTP}^{(10)}+E_{Das}$ binding energies, respectively. It must be noted that the experimental results are reproduced by the nonempirical models only when dispersion forces are accounted for. In the case of the computationally affordable $E_{EL,MTP}^{(10)}+E_{Das}$ model, the damped dispersion expression *E*_*Das*_ was included in the revised version, containing the halogen atom parameters. As for the empirical scoring, only GoldScore performance is adequate (*R* correlation coefficient of − 0.96) and comparable to either an approximate $E_{EL,MTP}^{(10)}+E_{Das}$ or the most robust *E*_*MP*2_ level of theory.

The performance achieved for the $E_{EL,MTP}^{(10)}+E_{Das}$ model with the revised *E*_*Das*_ parameters suggests that it could be successfully used in systems that involve halogenated species. It is especially important in the view of mostly unsatisfactory results obtained with the empirical scoring functions, which demonstrates that reliable description of such systems remains a challenge.

## Electronic supplementary material

Below is the link to the electronic supplementary material.

(PDF 344 KB)

## Footnotes

1 Unpublished results (2018)
