# Supplementary material for: Revisiting the halogen bonding between phosphodiesterase type 5 and its inhibitors
Source: J Mol Model. 2019 Jan 7;25(2):29. doi: 10.1007/s00894-018-3897-z (PMC6321839; doi:10.1007/s00894-018-3897-z)
Supplement: Supplementary file 1 — (PDF 344 KB) [file 894_2018_3897_MOESM1_ESM.pdf]

Journal of Molecular Modeling manuscript No.
(will be inserted by the editor)

Supplementary Material
Revisiting the halogen bonding between phosphodiesterase type 5 and its inhibitors.

Wiktoria Jedwabny · Edyta Dyguda-Kazimierowicz

Received: DD Month YEAR / Accepted: DD Month YEAR

Supplementary tables

The following results are provided:

- per-residue E_{MP2} binding energy (Table S1),
- scoring results obtained with empirical scoring functions (Table S2),
- comparison with the binding energy results provided by Ren et al [1] (Table S3).

Table S1 E_{MP2} interaction energy[a] per amino acid residues or residue pairs.

Residue	**5-I**	**5-Br**	**5-Cl**	**5-H**	**5-F**
Tyr612	-2.4	-2.0	-1.3	-1.8	-0.7
Asp764Leu765	-3.0	-1.6	-2.0	-2.8	-1.1
Ala767Ile768	-1.7	-1.4	-1.9	-0.3	-1.2
Gln775	-0.5	-0.4	-0.5	-0.7	-1.0
Ile778Ala779	-1.9	-0.9	-1.1	-2.0	-1.8
Val782	-3.0	-3.2	-3.2	-2.7	-2.6
Gln817	-14.5	-14.3	-14.6	-13.5	-13.4
Phe820	-6.4	-6.4	-5.4	-4.7	-4.9

[a] In units of $\mathrm{kcal \cdot mol^{-1}}$.

Comparison with the results of Ren et al [1]

Computational results presented in Ref. [1] include binding energy values characterizing interaction of three PDE5 inhibitors (**5-Cl**, **5-Br**, **5-I**) and Tyr612 as well as buried water molecule. Comparison of the results of Ren et al [1] and the corresponding values obtained in our study is provided in Table S3. The main conclusion put forward by Ren et al [1] involved the opposite trend regarding the strength of binding of **5-I** by Tyr612 residue and water molecule compared to the remaining two inhibitors. In particular, **5-I** complex is said to be characterized by stronger interaction with water molecule and weaker binding by Tyr612 residue, highlighting the significance of this particular buried water molecule for halogen bonding of **5-I** [1].

Such a conclusion is not supported by our results, wherein a nearby water molecule does not display any exceptional binding properties in the case of **5-I** complex (see Table S3). The interaction energy within **5-I**-water complex is the same as for the corresponding interaction present in **5-Cl**-water complex. The analogous interaction in the case of **5-Br** inhibitor appears to be of repulsive nature, presumably due to simplified optimization protocol employing force field description.

The main difference regarding the computational models employed by Ren et al [1] with respect to our current work consists in optimization of the entire protein-ligand complexes prior to QM calculations (see the main text for the optimization protocol). In our opinion, the initial optimization is required to alleviate the structural deficiencies resulting from insufficient resolution of crystal structures (e.g., 2.44 Å for the PDE5-**5-I** complex). As shown in Fig. S1, optimization of the PDE5-**5-I** complex introduced some differences in the protein structure, and the calculated root mean square deviation between the two complexes was equal to 0.55 Å. Importantly, the hydrogen and halogen bond network connecting inhibitor **5-I**, Tyr612 residue and water (HOH) molecule changed upon optimization.

W. Jedwabny
Department of Chemistry, Wrocław University of Science and Technology, Wrocław, Poland

E. Dyguda-Kazimierowicz
Department of Chemistry, Wrocław University of Science and Technology, Wrocław, Poland
E-mail: Edyta.Dyguda@pwr.edu.pl

Table S2 The performance of empirical scoring.

Inhibitor	pK_d [a]	XBSF [b]	ChemPLP$_P$ [b]	PLP [b]	GoldScore [c]	ChemScore [c]	ASP [c]	ChemPLP$_G$ [c]	GlideSP [b]	GlideXP [b]
**5-I**	6.82	-4.7	-56.0	-50.1	33.3	18.4	23.2	35.0	-7.1	-7.7
**5-Br**	6.38	-4.3	-54.2	-50.4	30.5	18.0	22.3	29.8	-7.2	-7.6
**5-Cl**	6.12	-5.2	-56.9	-51.4	29.7	18.9	23.4	37.9	-7.8	-8.1
**5-H**	5.84	-4.3	-51.5	-47.0	25.2	16.8	22.0	32.7	-7.0	-7.1
**5-F**	5.76	-4.9	-54.2	-49.6	26.2	16.7	21.8	32.7	-7.5	-7.7
R [d]		+0.05	-0.54	-0.44	-0.96	-0.68	-0.66	-0.10	+0.28	-0.25
N_{pred} [e]		50.0	70.0	60.0	90.0	80.0	80.0	50.0	40.0	50.0

[a] pK_d values are taken from Ref. 1.
[b] In $kcal \cdot mol^{-1}$.
[c] In arbitrary units.
[d] Correlation coefficient between given scoring function and the experimental inhibitory activity.
[e] Percentage of successful predictions [%].

Furthermore, our results were obtained with a larger basis set, def2TZVP, and the more robust QM method, MP2, while Ren et al [1] reported the results of M06-2X/6-311+G(d) calculations. Overall, based on the results acquired with a more reliable computational protocol accounting for structure optimization and better QM description, we conclude that the water molecule positioned in the vicinity of 5-substituent does not seem to significantly contribute to halogen bonding of PDE5 inhibitors, including **5-I** compound.

Table S3 Binding energy values[a] for selected interacting moieties of PDE5-inhibitor complexes.

	**5-Cl**		**5-Br**		**5-I**	
	MP2 [b]	DFT [c]	MP2	DFT	MP2	DFT
HOH	-0.2	-0.1	+1.1	-0.3	-0.2	-3.2
Tyr612	-1.3	-1.2	-2.0	-2.1	-2.4	-1.3

[a] In units of $kcal \cdot mol^{-1}$.
[b] MP2/def2TZVP results obtained in the current study.
[c] M06-2X/6-311+G(d) results reported by Ren et al [1].

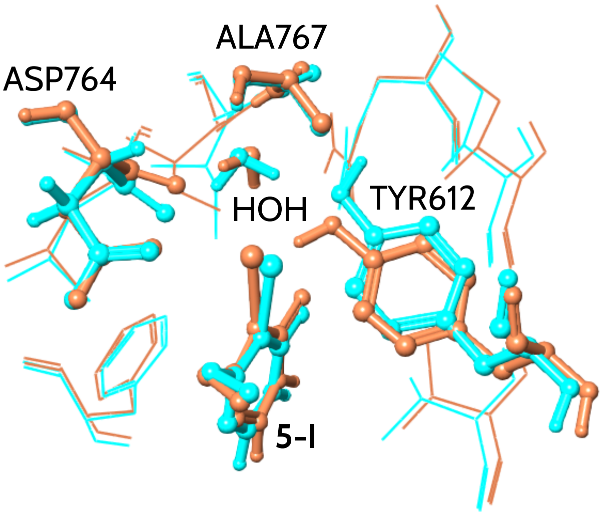

Fig. S1 Comparison of the PDE5–**5-I** complex before (orange) and after (turquoise) optimization. The residues and buried water molecule that constitute the simplified binding site reported by Ren et al [1] are given in ball-and-stick representation.

References

1. Ren J, He Y, Chen W, Chen T, Wang G, Wang Z, Xu Z, Luo X, Zhu W, Jiang H, Shen J, Xu Y (2014) Thermodynamic and structural characterization of halogen bonding in protein-ligand interactions: A case study of PDE5 and its inhibitors. J Med Chem 57(8):3588–3593
